# Therapeutic Potential of the Intestinal Microbiota for Immunomodulation of Food Allergies

**DOI:** 10.3389/fimmu.2020.01853

**Published:** 2020-08-14

**Authors:** Luisa Kreft, Christian Hoffmann, Caspar Ohnmacht

**Affiliations:** ^1^Mucosal Immunology Group, Center of Allery and Environment (ZAUM), Technical University and Helmholtz Center Munich, Munich, Germany; ^2^Member of the German Center of Lung Research (DZL), Munich, Germany; ^3^Department of Food Science and Experimental Nutrition, Food Research Center (FoRC), School of Pharmaceutical Sciences, University of São Paulo, São Paulo, Brazil

**Keywords:** intestinal microbiota, food allergy, regulatory T cells, Foxp3, oral tolerance, anaphylaxis, bacterial metabolites

## Abstract

Food allergy is an atopic disease that is caused by the immune system targeting harmless food antigens that can result in life-threatening anaphylaxis. As humans and microbes have co-evolved, inevitably commensal microbes have a tremendous impact on our health. As such, the gut with its enormous microbial richness reflects a highly tolerogenic environment at steady state, in which immune cells are educated to react in a well-calibrated manner to food and microbial antigens. Recent evidence indicates that the susceptibility to food allergy is critically linked to microbial dysbiosis and can be transmitted by microbial transfer from humans to mice. Experimental work and epidemiological studies further point toward a critical time window in early childhood during which the immune system is imprinted by microbial colonization. Particularly, Foxp3-expressing regulatory T cells turn out to be key players, acting as rheostats for controlling the magnitude of food allergic reactions. An increasing number of bacterial metabolites has recently been shown to regulate directly or indirectly the differentiation of peripherally induced Tregs, most of which co-express the RAR-related orphan receptor gamma t (RORγt). Genetic ablation provided additional direct evidence for the importance of RORγt+ Tregs in food allergy. Future strategies for the stratification of food allergic patients with the aim to manipulate the intestinal microbiota by means of fecal transplantation efforts, pre- or probiotic regimens or for boosting oral immunotherapy may improve diagnosis and therapy. In this review some of the key underlying mechanisms are summarized and future directions for potential microbial therapy are explored.

## Introduction

The enormous collection of microorganisms living in and on us is collectively referred to as microbiota. Bacteria, archaea, eukaryotes, and their associated viruses compose a highly complex microbial ecosystem (1). The microbiota has co-evolved during the evolution of all multicellular organisms and has become a *de facto* and even necessary “organ” in all modern vertebrates, fulfilling basic functions like the provision of nutrients and essential vitamins (2, 3). Today, there is strong evidence that correct physiological functioning of this organ is dependent on a harmonious host-microbiota relationship (4).

On the other hand, vertebrates have evolved complex innate and adaptive immune functions, responsible for detecting, containing, and eliminating a large array of microbial pathogens (5, 6). Even some of the simplest forms of multicellular organisms, such as hydrozoans, exhibit innate immune pathways responsible for the recognition, and maintenance of certain bacterial associations (7). The discrimination between beneficial and pathogenic microorganisms poses a major challenge for the immune system that we are only beginning to understand.

**Figure 1:**
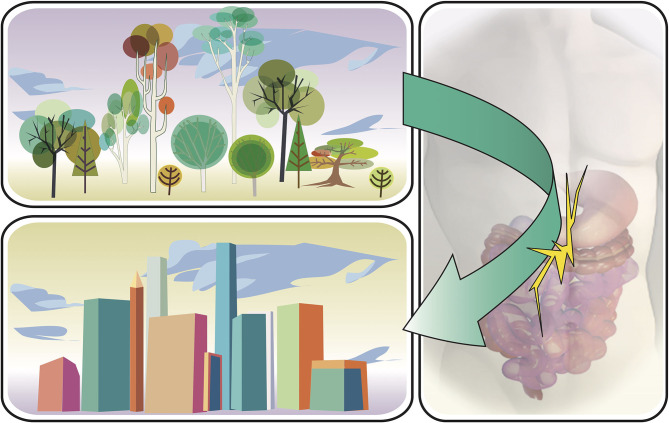
Illustration of changes in life style factors having an impact on the intestinal microbiome. Humans have shifted very quickly from an ancestral to a modern way of living, evolutionarily speaking. These changes have impacted the balance between the intestinal microbiota and the immune system.

Humans are no different in this respect: we have been surrounded by a great number of microorganisms for the majority of human history co-evolving with our microbiota (1). Today, abundant evidence indicates that the microbiota is essential for the correct functioning of human physiology (8). However, recent human development has shifted our relationship with microorganisms in a short time period, evolutionarily speaking, and these rapid changes were not accompanied by the necessary adaptations to our changing microbiota (Figure 1). Improvements in our way of life have extended our life spans, through medicine, sanitation, and industrialization of our food production system. With these changes, a large array of microbial infections is no longer a death sentence for humanity. Simultaneously, there was a marked increase in prevalence of several immune-related disorders, such as Crohn's disease, asthma, and food allergies (9). This relationship was already noticed in the late 1980's and early 1990's, eventually being named the Hygiene Hypothesis: we removed the infections, but in the process, the immune system lost something as well (10).

Today, we understand that not the removal of pathogens itself, as initially proposed, has an effect on our immune responses, but the processes that eliminated dangerous pathogens have also eliminated other microbial and fungal bystanders and eventually multicellular parasites such as helminths (e.g., *Ascaris, Trichuris*, or *Schistosomes*) from our microbiota, breaking a beneficial relationship selected over eons of evolution (11). Eventually, modern humans and particularly those living in developed countries have irretrievably lost certain ancient microorganisms that have been instrumental to set up a healthy host-microbial homeostasis (12). If this homeostasis cannot adequately be reached during colonization in childhood, an enhanced risk of atopic diseases including food allergy may be one long-term consequence.

## Alteration of the Gut Microbiota in Food Allergic Patients

Some studies have directly related food allergies and the gut microbiome in patients (13, 14). Still, the current understanding for the implication of the microbiota in human allergies is mostly based on epidemiological studies including birth cohorts. The current use of bacterial 16S rRNA sequencing, as well as metagenomic sequencing, allows the characterization of the microbial composition of both the environment and the intestinal microbiome. Several large cohort studies conducted in children raised in a traditional farming environment revealed a protective effect of this environment and this has been positively correlated to bacterial and fungal diversities found in these environments (15–19). Similarly, distinct communities of bacteria have been found in stool samples of neonates which preceded later signs of allergic asthma (20). Importantly, such communities led to an enhanced allergic lung inflammation after transplantation into germfree mice that provided direct evidence for the underlying causality. In another study, analysis of stool samples from neonates revealed three different microbial patterns one of which being associated with multiple sensitizations to various allergens: such microbiomes showed lower relative abundance of *Bifidobacterium, Akkermansia*, and *Faecalibacterium* and increased levels of the metabolite 12,13-dihydroxy-9Z-octadecenoic acid (12,13-DiHome), which has been confirmed to aggravate allergic lung inflammation in murine models (21, 22).

In prior studies, the amount of exposure to conserved bacterial products such as Lipopolysaccharide (LPS) from the environment has already been negatively correlated to atopic sensitization and this was associated with reduced production of cytokines by peripheral blood leukocytes (16). Another more recent large cohort study tracked the intestinal microbiome composition within the first years of life. Although initially intended for observing the microbiome's influence on the incidence of diabetes, the cohort provided the opportunity to show HLA matched class risks in geographically distinct locations (23). Despite the initial non-allergic focus of the study, it found that growing up with a comparatively modern lifestyle is associated with an increased risk of atopy and autoimmune manifestations. Children from Finland showed a relatively high sensitization rate toward typical allergens such as milk and egg, Russian children only showed a relatively low frequency of sensitization and Estonian children were intermediate. Fecal samples of Finish and Estonian children showed a relative high abundance of *Bacteroides*, while Russian children had a higher abundance of *Bifidobacterium*. Interestingly, metagenomic analysis revealed that LPS synthesizing gene clusters were among the most differentially expressed pathways between the three groups. The authors then demonstrated that the origin of the LPS (*Bacteroides* spp. vs. *E. coli*) leads to a structural difference with a strong effect on the immunostimulatory capacity of these variants on primary human peripheral blood monocytes (23). This revealed how the structural variation of a single component sensed by pattern recognition receptors (PRRs) due to differences of microbial compositions early in life may contribute to enhanced sensitization rates and thus risk of food allergy in children. It is possible that much more structural variations of such microbial determinants sensed by the PRRs of the immune system exist and educate the microbiota-host homeostasis in children.

## Mechanistic Insights From Murine Models

While human studies are often limited due to being correlative, murine models provide a defined system for the determination of the mechanistic basis of the microbiota's impact on health and disease in the host. However, it has to be acknowledged that there are profound differences between the digestive system and microbiota of humans and mice as reviewed in (24).

Experimental evidence from murine models has shown that the intestinal microbiota protects the host from allergic inflammation by: (I) contributing to the establishment of antigen-specific oral tolerance, (II) preventing excessive inflammation, (III) impacting on various aspects of host physiology, such as intestinal barrier function, degradation of xenobiotics, production of metabolites, and, (IV) directly contributing to the development of both the innate and adaptive immune system, such as basophil hematopoiesis (25).

Mice housed under germ-free conditions or treated with broad-spectrum antibiotics gave the first direct hints that the microbiota is essential to maintain a balanced (and thus a non-type 2 immunity prone) immune system. Such mice typically show elevated serum Immunoglobulin E (IgE) levels while all other immunoglobulins are downregulated (25–27). The microbiota maintains IgE at basal levels which requires microbial exposure during early live (28). In the absence of microbial colonization during this time window, Th2-skewed follicular helper T cells (Tfh) may develop that support class-switching to IgE in B cells which is directed against food antigens (29). A recent study has identified a special subset of allergen-specific Tfh cells that secrete IL-4 and particularly IL-13 which are instrumental to induce anaphylactic IgE (30). Interestingly, another study found expression of RAG proteins in B cells within the intestinal lamina propria exclusively around the time of weaning (31). As the positive and negative selection of B cells is thought to occur primarily in the bone marrow these results point toward another step of B cell education in response to microbial colonization. This could be particularly important in the case of food allergy as IgE specific for both bacteria and food antigens has been found in patients and mice with food allergy (32).

Antibiotic treatment in mice can also result in exaggerated basophil-mediated Th2 cell responses and allergic inflammation, indicating that the microbiota directly restrains the size of circulating basophil populations by limiting the proliferation of bone marrow resident precursor populations (25). Recolonization of germfree mice with specific bacterial strains (such as *Clostridia* mixtures) leads to decreased allergen-specific IgE and reduced susceptibility to anaphylactic reactions in a model of peanut allergy (33). Recolonization with *Clostridia* in this model also reduced the uptake of the allergen by affecting the intestinal barrier permeability. This effect can be mimicked by genetically limiting intestinal barrier integrity, e.g., through knockout of the transcription factor retinoic acid receptor-related orphan receptor gamma t (RORγt). RORγt is a major regulator of intestinal IL-22-producing immune cells such as ILC3s or γδ T cell subsets known to enforce the intestinal barrier (33, 34). Still, permeability in the intestinal tract is most likely not the only decisive factor as exposure to intestinal microbes generally leads rather to a Th1- and Th17-dominated immune response.

Furthermore, transplantation of gut microbiota samples from children with food allergy into germfree animals led to more severe anaphylactic reactions when such xenotransplanted mice were challenged with allergen in a food allergy model (32, 35). This provides direct evidence that a human intestinal microbiota from allergic children can confer this susceptibility to another species, representing a key step in understanding the underlying causality. Other barrier sites apart from the intestine also show exaggerated allergic reactions in the absence of microorganisms. For example, germfree mice or very young mice with incomplete colonization show an increased Th2 immunity and worsened allergic lung inflammation, supporting the beneficial role of the microbiota also in the lung (27, 36).

## The Microbiota and Tregs

Regulatory T cells (Tregs), characterized by the expression of the transcription factor Foxp3, and Tr1 cells, characterized by the expression of immunoregulatory cytokines such as IL-10 in the absence of Foxp3, are critical for regulating immune responses, dampening inflammation and for general homeostasis of barrier surfaces (37, 38). The microbiota can directly impact on the frequency of Tregs, as oral administration of murine and human *Clostridia* strains transferred into germfree mice leads to a drastic increase in Treg frequencies within the colon (39, 40). Surprisingly, germfree mice do not harbor less Tregs in the small intestine which may be due to an altered de-differentiation process of Tregs at this site (41). Similar to the intestinal microbiota, colonization of the skin was shown to recruit Tregs during a specific time window and these Tregs are most likely specific for such commensal microbes (42).

Two types of Tregs can be distinguished according to the origin of differentiation: Thymic-derived Tregs (tTregs) that are selected within the thymus probably due to recognition of self-antigens, and peripherally induced Tregs (pTregs) that differentiate in peripheral organs from naïve T cells. In the intestine, microbial colonization is responsible for inducing the differentiation of pTregs (43). Preventing pTreg differentiation by knocking out the CNS1 (conserved non-coding sequence 1) region next to the Foxp3 promotor revealed that these immune cells prevent a spontaneous type 2 immune bias at mucosal sites (44). Dietary antigens from solid foods are also tolerized by inducing a population of short-lived pTregs in the small intestine where uptake of nutrients including food allergens most likely takes place (45). Interestingly, germfree animals raised in the absence of macronutrients revealed that in the absence of both dietary and microbial antigens (and therefore the majority of intestinal pTregs), the adaptive intestinal T cell response is heavily skewed toward a Th1-dominated response whereas the absence of microbes alone heavily skews the intestinal T cell responses toward a Th2-dominated and therefore pro-food allergy immune response (45). Therefore, immunological tolerance of dietary antigens is of pivotal importance but the intestinal microbiome most likely is a key driver in preventing the Th2-skewing after recognition of dietary antigens and limiting the susceptibility toward food allergy. We and others have demonstrated that microbiota-induced pTregs share features with intestinal Th17 cells, such as the expression of the transcription factor RORγt (46, 47). Their induction can be mediated by a diverse range of bacterial species and the lack of RORγt+ Tregs leads to exacerbated Th2 and Th17 pathology in the intestine (46, 47). Noteworthy, RORγt+ Tregs have also been detected upon oral exposure to food antigens using transgenic T cells recognizing an epitope from chicken ovalbumin (45, 46). Due to the highly artificial nature of such T cell receptor (TCR) transgenic T cell transfer approaches and the observation that germfree mice raised in the presence of solid food and thus food antigens still show a dramatic reduction in RORγt+ Tregs, the relevance of this observation remains to be investigated in more physiological conditions.

In order to exploit the induction of pTregs by the microbiota for therapeutic purposes, the underlying mechanisms for this induction needs to be understood on a molecular level. Recent evidence indicates that bacterial metabolites, such as short-chain fatty acids (SCFAs) (48, 49) and cell surface polysaccharides from typical commensals, such as *Bifidobacterium bifidum*, are capable of inducing pTregs (50), further confirming the impact of the microbiota on this cell population. More recently, secondary bile acids were shown to induce Foxp3 expression in naïve T cells either directly or in a dendritic cell-dependent manner (51–53). In particular, Isodeoxycholic acid producing bacteria increased colonic RORγt+ Tregs *in vivo*, which was not observed when transplanting bacteria unable to generate this secondary bile acid (53).

Furthermore, RORγt+ Tregs have been shown to have a protective role in a model of food allergy, and the expression of RORγt is indispensable for this function (32). The same group reported that RORγt-expression in Tregs can also be detrimental for allergic inflammation of the lung, as it may drive the expression of pro-inflammatory cytokines in different conditions (54). These studies relied on an elegant murine model of enhanced signaling via the interleukin 4 receptor (IL-4R). It is based on a point mutation within the intracellular domain of the IL-4R that has also been found in a subset of patients with food allergy (55). As a consequence, intestinal Tregs start to (over-)express the transcription factor Gata3 and secrete the cytokine IL-4, making this Treg population rather a pathogenic driver of food allergy than an immune regulator (56). Such type 2 prone Tregs are less stable but most likely their differentiation is independent from microbiota effects, as Gata3+ Tregs can be found in germfree animals and Gata3 and RORγt expression are usually mutually exclusive (46, 57). Still, transplantation of fecal samples from IL-4R mutated mice subjected to food allergy can confer the enhanced Th2 skewing and allergic reactions to wildtype animals suggesting that excessive IL-4R signaling also has a strong impact on the microbiota (58). In other contexts, Gata3 expression in Tregs has been proposed as a general hallmark of Tregs residing within different tissues as compared to secondary lymphoid organs making it questionable whether Gata3+ Tregs can generally be considered pathogenic in patients without mutations in the IL-4R pathway (59).

**Figure 2:**
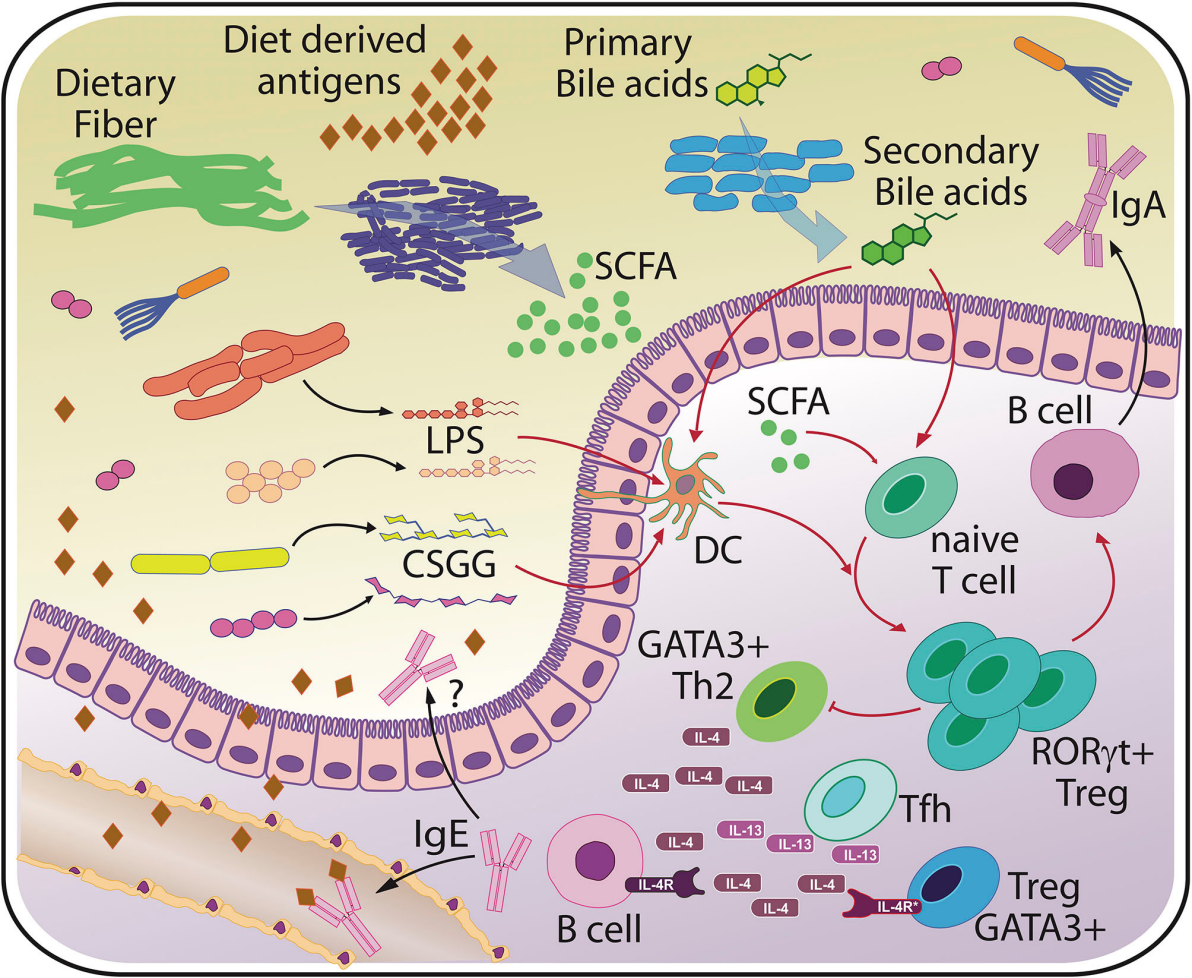
Basic principles of host-microbiota interaction relevant for food allergy. The scheme indicates major bacterial molecules from various bacterial sources that have been linked to (RORγt+) Treg induction and protection from sensitization and/or food allergy. Protective microbial factors may include but are not limited to variants of lipopolysaccharide (LPS) from different bacterial species, cell surface polysaccharides from typical commensals such as *Bifidobacterium bifidum* (CSGG, Cell-surface β-glucan/galactan polysaccharides), short chain fatty acids (SCFA) and secondary bile acids that all act directly on T cells or on accessory cells such as dendritic cells (DC). Microbiota-dependent RORγt+ Tregs are thought to protect against excessive accumulation of T cells secreting type 2 cytokines such as interleukin 4 (IL-4) and interleukin 13 (IL-13). Ultimately, a tight restriction of B cells secreting IgE specific for food and bacterial derived antigens, which is thought to be mediated by T follicular helper cells (Tfh), must be achieved to prevent systemic reactions, such as anaphylaxis. Regulation of the intestinal microbiota may be accomplished through bacterial coating by host or maternally-derived luminal IgA and controlled barrier function.

Altogether, the discovery of different Tregs subsets with unique functions offers a cellular and molecular link to how microbial compositions may modulate the risk for allergic inflammation (Figure 2). Whether these microbial effects act directly on T cells, and how much other cellular players known to regulate T cell fate, such as dendritic cells, contribute to microbiota-mediated effects remains a matter of current investigation (53, 60).

## Discussion

Current treatment for food allergies mainly comprises rescue medication treatment after exposure and allergen avoidance. Desensitization can only be achieved with specific oral immunotherapy (OIT), however it is a lengthy process of several years and full remission only occurs in up to 50% of the subjects who have undergone OIT for milk, egg and peanut and adverse reactions are common (61). Treatment options utilizing a preventive effect of the microbiota have been few and limited to probiotics and fecal microbial transplantations (FMTs) with no clear overall effective results (62). The integration of RORγt+ Treg frequencies for patient stratification and assessment of OIT and FMT effectiveness could lead to better success rates in the future. As more and more bacterial strains and bacterial metabolites have been identified to regulate RORγt+ Treg homeostasis, more patient-tailored treatment options may be developed. For instance, colonization of mice and humans with certain *Clostridiales* taxa has already been linked to protection from food allergy, yet the efficacy of a therapeutic application in humans remains to be investigated (32, 35). Furthermore, the clinical application of microbe-based therapeutics may be complicated by several factors including the variability of the host microbiome composition in FMTs, stably imprinted intestinal microbial ecosystems, unforeseen side effects on host physiology and the impossibility to reprogram existing long-lived immune cells (e.g. T-and plasma B cell populations). However, not only microbe- but also parasite-derived molecules may be used in the future for the treatment of food allergies as particularly helminth parasites have been part of our intestinal microbiota over long periods of human evolution. Currently, such treatments are already tested for therapeutic purposes in allergic airway inflammation models and it has already been established that they can act on several cell types, including myeloid cells, and on the differentiation/proliferation of Tregs (63–65).

In general, RORγt+ Tregs may serve as an indicator for the patient's microbiota in a “dysbiotic” state and the respective patient being at elevated risk of allergic or other inflammatory diseases (66). Still, the assessment of this cellular parameter is currently not possible in clinical settings and exemplifies the medical need for the identification of surrogate measurements. As long as the microbiota host interaction and the metabolic pathways of certain species remain only partially understood, the interventions to manipulate the microbiota will remain largely unspecific, and the potential of such microbiota-based therapies are limited. Ultimately, the exploitation of metabolic pathways and other RORγt+ Treg-stimulating agents by changing microbial compositions to enhance oral tolerance and protect from sensitization and food allergy is nevertheless highly attractive. Many questions in this regard remain unanswered, for example, how the generic induction of (RORγt+) Tregs by microbial consortia or their products can induce an antigen- or allergen-specific tolerogenic B and T cell response. Alternatively, a polyclonal and/or unspecific TCR repertoire within the Treg population may be sufficient to suppress allergen specific Th2 cells in a bystander suppression or by acting on accessory cells, e.g., dendritic cells. One may ask why evolution did not select for a general higher level of (RORγt+) Tregs to avoid overt intestinal inflammation and the risk of food allergy. It is perhaps because generalized suppression of adaptive immunity at mucosal sites through excessive pTreg generation could be dangerous, as contact with facultative pathogens are frequent and risk of infections are high. Setting the correct bar for RORγt+ Tregs and homeostasis with the developing microbiota needs to occur in childhood, and seems to also be imprinted from the mother to the next generation by a double-negative loop involving maternally transmitted IgA (67, 68). While this mechanism ensures that the next generation benefits from a maternal experience on the correct bar for a beneficial host-microbiota equilibrium, microbial adaptations are still able to fine-tune the level of RORγt+ Tregs throughout life and offer therapeutic options. Thus, a well-calibrated balance between pro- and anti-inflammatory signals needs to be integrated by accessory cells such as dendritic cells, or even directly by activated T cells at the right time point of colonization. One long-term goal is therefore to better understand this complex integration in order to combine this knowledge with OIT regimens or for diagnostic purposes to determine food allergic risk patterns ideally already early in life.

## Author Contributions

All authors contributed to the writing of this review and have read and approved the final version.

## Conflict of Interest

The authors declare that the research was conducted in the absence of any commercial or financial relationships that could be construed as a potential conflict of interest.

## References

[1] Ley RE Lozupone CA Hamady M Knight R Gordon JI. Worlds within worlds: evolution of the vertebrate gut microbiota. Nat Rev Microbiol. (2008) 6:776–88. 10.1038/nrmicro1978 18794915 PMC2664199

[2] Baquero F Nombela C The microbiome as a human organ. Clin Microbiol Infect. (2012) 18:2–4. 10.1111/j.1469-0691.2012.03916.x 22647038

[3] Sherrill-Mix S McCormick K Lauder A Bailey A Zimmerman L Li Y. Allometry and ecology of the bilaterian gut microbiome. mBio. (2018) 9:e00319–18. 10.1128/mBio.00319-18 29588401 PMC5874926

[4] Lozupone CA Stombaugh JI Gordon JI Jansson JK Knight R. Diversity, stability and resilience of the human gut microbiota. Nature. (2012) 489:220–30. 10.1038/nature11550 22972295 PMC3577372

[5] Cooper MD Herrin BR. How did our complex immune system evolve? Nat Rev Immunol. (2010) 10:2–3. 10.1038/nri2686 20039476

[6] Voogdt CGP van Putten JPM Chapter 13 - The evolution of the toll-like receptor system. In: Malagoli D, edior. The Evolution of the Immune System. San Diego: Academic Press (2016). p. 311–30. 10.1016/B978-0-12-801975-7.00013-X

[7] Franzenburg S Fraune S Kunzel S Baines JF Domazet-Loso T Bosch TCG. MyD88-deficient hydra reveal an ancient function of TLR signaling in sensing bacterial colonizers. Proc Natl Acad Sci USA. (2012) 109:19374–9. 10.1073/pnas.1213110109 23112184 PMC3511086

[8] Martin AM Sun EW Rogers GB Keating DJ. The influence of the gut microbiome on host metabolism through the regulation of gut hormone release. Front Physiol. (2019) 10:428. 10.3389/fphys.2019.00428 31057420 PMC6477058

[9] Platts-Mills TAE. The allergy epidemics: 1870-2010. J Allergy Clin Immunol. (2015) 136:3–13. 10.1016/j.jaci.2015.03.048 26145982 PMC4617537

[10] Strachan DP. Hay fever, hygiene, and household size. BMJ. (1989) 299:1259–60. 10.1136/bmj.299.6710.1259 2513902 PMC1838109

[11] Scudellari M. Cleaning up the hygiene hypothesis. Proc Natl Acad Sci USA. (2017) 114:1433–6. 10.1073/pnas.1700688114 28196925 PMC5320962

[12] Blaser MJ Falkow S. What are the consequences of the disappearing human microbiota? Nat Rev Microbiol. (2009) 7:887–94. 10.1038/nrmicro2245 19898491 PMC9354563

[13] Hua X Goedert JJ Pu A Yu G Shi J. Allergy associations with the adult fecal microbiota: analysis of the american gut project. EBioMedicine. (2016) 3:172–9. 10.1016/j.ebiom.2015.11.038 26870828 PMC4739432

[14] Ling Z Li Z Liu X Cheng Y Luo Y Tong X. Altered fecal microbiota composition associated with food allergy in infants. Appl Environ Microbiol. (2014) 80:2546–54. 10.1128/AEM.00003-14 24532064 PMC3993190

[15] Stein MM Hrusch CL Gozdz J Igartua C Pivniouk V Murray SE. Innate immunity and asthma risk in amish and hutterite farm children. N Engl J Med. (2016) 375:411–21. 10.1056/NEJMoa1508749 27518660 PMC5137793

[16] Braun-Fahrländer C Riedler J Herz U Eder W Waser M Grize L. Environmental exposure to endotoxin and its relation to asthma in school-age children. N Engl J Med. (2002) 347:869–77. 10.1056/NEJMoa020057 12239255

[17] Ege MJ Strachan DP Cookson WOCM Moffatt MF Gut I Lathrop M. Gene-environment interaction for childhood asthma and exposure to farming in Central Europe. J Allergy Clin Immunol. (2011) 127:138–44.e4. 10.1016/j.jaci.2010.09.041 21211648

[18] Ege MJ Frei R Bieli C Schram-Bijkerk D Waser M Benz MR. Not all farming environments protect against the development of asthma and wheeze in children. J Allergy Clin Immunol. (2007) 119:1140–7. 10.1016/j.jaci.2007.01.037 17349684

[19] Ege MJ Mayer M Normand AC Genuneit J Cookson WOCM Braun-Fahrländer C. Exposure to environmental microorganisms and childhood asthma. N Engl J Med. (2011) 364:701–9. 10.1056/NEJMoa1007302 21345099

[20] Arrieta M-C Stiemsma LT Dimitriu PA Thorson L Russell S Yurist-Doutsch S. Early infancy microbial and metabolic alterations affect risk of childhood asthma. Sci Transl Med. (2015) 7:307ra152. 10.1126/scitranslmed.aab2271 26424567

[21] Fujimura KE Sitarik AR Havstad S Lin DL Levan S Fadrosh D. Neonatal gut microbiota associates with childhood multisensitized atopy and T cell differentiation. Nat Med. (2016) 22:1187–91. 10.1038/nm.4176 27618652 PMC5053876

[22] Levan SR Stamnes KA Lin DL Panzer AR Fukui E McCauley K Elevated faecal 12,13-diHOME concentration in neonates at high risk for asthma is produced by gut bacteria and impedes immune tolerance. Nat Microbiol. (2019) 4:1851–61. 10.1038/s41564-019-0498-2 31332384 PMC6830510

[23] Vatanen T Kostic AD D'Hennezel E Siljander H Franzosa EA Yassour M Variation in microbiome LPS immunogenicity contributes to autoimmunity in humans. Cell. (2016) 165:842–53. 10.1016/j.cell.2016.04.007 27133167 PMC4950857

[24] Hugenholtz F de Vos WM. Mouse models for human intestinal microbiota research: a critical evaluation. Cell Mol Life Sci. (2018) 75:149–60. 10.1007/s00018-017-2693-8 29124307 PMC5752736

[25] Hill DA Siracusa MC Abt MC Kim BS Kobuley D Kubo M. Commensal bacteria-derived signals regulate basophil hematopoiesis and allergic inflammation. Nat Med. (2012) 18:538–46. 10.1038/nm.2657 22447074 PMC3321082

[26] McCoy KD Harris NL Diener P Hatak S Odermatt B Hangartner L. Natural IgE production in the absence of MHC class II cognate help. Immunity. (2006) 24:329–39. 10.1016/j.immuni.2006.01.013 16546101

[27] Herbst T Sichelstiel A Schär C Yadava K Bürki K Cahenzli J. Dysregulation of allergic airway inflammation in the absence of microbial colonization. Am J Respir Crit Care Med. (2011) 184:198–205. 10.1164/rccm.201010-1574OC 21471101

[28] Cahenzli J Köller Y Wyss M Geuking MB McCoy KD. Intestinal microbial diversity during early-life colonization shapes long-term IgE levels. Cell Host Microbe. (2013) 14:559–70. 10.1016/j.chom.2013.10.004 24237701 PMC4049278

[29] Hong SW Eunju O Lee JY Lee M Han D Ko HJ. Food antigens drive spontaneous IgE elevation in the absence of commensal microbiota. Sci Adv. (2019) 5:eaaw1507. 10.1126/sciadv.aaw1507 31131325 PMC6531000

[30] Gowthaman U Chen JS Zhang B Flynn WF Lu Y Song W. Identification of a T follicular helper cell subset that drives anaphylactic IgE. Science. (2019) 365:eaaw6433 10.1126/science.aaw6433 31371561 PMC6901029

[31] Wesemann DR Portuguese AJ Meyers RM Gallagher MP Cluff-Jones K Magee JM. Microbial colonization influences early B-lineage development in the gut lamina propria. Nature. (2013) 501:112–5. 10.1038/nature12496 23965619 PMC3807868

[32] Abdel-Gadir A Stephen-Victor E Gerber GK Noval Rivas M Wang S Harb H Microbiota therapy acts via a regulatory T cell MyD88/RORγt pathway to suppress food allergy. Nat Med. (2019) 25:1164–74. 10.1038/s41591-019-0461-z 31235962 PMC6677395

[33] Stefka AT Feehley T Tripathi P Qiu J McCoy K Mazmanian SK. Commensal bacteria protect against food allergen sensitization. Proc Natl Acad Sci USA. (2014) 111:13145–50. 10.1073/pnas.1412008111 25157157 PMC4246970

[34] Ohnmacht C. Tolerance to the intestinal microbiota mediated by ROR(γt) + cells. Trends Immunol. (2016) 37:477–86. 10.1016/j.it.2016.05.002 27255270

[35] Feehley T Plunkett CH Bao R Choi Hong SM Culleen E Belda-Ferre P. Healthy infants harbor intestinal bacteria that protect against food allergy. Nat Med. (2019) 25:448–53. 10.1038/s41591-018-0324-z 30643289 PMC6408964

[36] Gollwitzer ES Saglani S Trompette A Yadava K Sherburn R McCoy KD. Lung microbiota promotes tolerance to allergens in neonates via PD-L1. Nat Med. (2014) 20:642–7. 10.1038/nm.3568 24813249

[37] Josefowicz SZ Lu LF Rudensky AY. Regulatory T Cells: mechanisms of differentiation and function. Annu Rev Immunol. (2012) 30:531–64. 10.1146/annurev.immunol.25.022106.141623 22224781 PMC6066374

[38] Roncarolo MG Gregori S Bacchetta R Battaglia M Gagliani N. The biology of t regulatory type 1 cells and their therapeutic application in immune-mediated diseases. Cell Press. (2018) 49:1004–19. 10.1016/j.immuni.2018.12.001 30566879

[39] Atarashi K Tanoue T Shima T Imaoka A Kuwahara T Momose Y. Induction of colonic regulatory T cells by indigenous *Clostridium* species. Science. (2011) 331:337–41. 10.1126/science.1198469 21205640 PMC3969237

[40] Atarashi K Tanoue T Oshima K Suda W Nagano Y Nishikawa H. Treg induction by a rationally selected mixture of Clostridia strains from the human microbiota. Nature. (2013) 500:232–6. 10.1038/nature12331 23842501

[41] Mucida D Park Y Kim G Turovskaya O Scott I Kronenberg M. Reciprocal TH17 and regulatory T cell differentiation mediated by retinoic acid. Science. (2007) 317:256–60. 10.1126/science.1145697 17569825

[42] Scharschmidt TC Vasquez KS Truong HA Gearty SV Pauli ML Nosbaum A. A wave of regulatory T cells into neonatal skin mediates tolerance to commensal microbes. Immunity. (2015) 43:1011–21. 10.1016/j.immuni.2015.10.016 26588783 PMC4654993

[43] Geuking MB Cahenzli J Lawson MAE Ng DCK Slack E Hapfelmeier S. Intestinal bacterial colonization induces mutualistic regulatory T cell responses. Immunity. (2011) 34:794–806. 10.1016/j.immuni.2011.03.021 21596591

[44] Josefowicz SZ Niec RE Kim HY Treuting P Chinen T Zheng Y. Extrathymically generated regulatory T cells control mucosal Th2 inflammation. (2012) 482:395–9. 10.1038/nature10772 22318520 PMC3485072

[45] Kim KS Hong SW Han D Yi J Jung J Yang BG. Dietary antigens limit mucosal immunity by inducing regulatory T cells in the small intestine. Science. (2016) 351:858–63. 10.1126/science.aac5560 26822607

[46] Ohnmacht C Park JH Cording S Wing JB Atarashi K Obata Y. The microbiota regulates type 2 immunity through RORγt+ T cells. Science. (2015) 349:989–93. 10.1126/science.aac4263 26160380

[47] Sefik E Geva-Zatorsky N Oh S Konnikova L Zemmour D McGuire AM. Individual intestinal symbionts induce a distinct population of RORγ+ regulatory T cells. Science. (2015) 349:993–7. 10.1126/science.aaa9420 26272906 PMC4700932

[48] Smith PM Howitt MR Panikov N Michaud M Gallini CA Bohlooly YM. The microbial metabolites, short-chain fatty acids, regulate colonic T reg cell homeostasis. Science. (2013) 341:569–73. 10.1126/science.1241165 23828891 PMC3807819

[49] Arpaia N Campbell C Fan X Dikiy S van der Veeken J deRoos P. Metabolites produced by commensal bacteria promote peripheral regulatory T cell generation. Nature. (2013) 504:451–5. 10.1038/nature12726 24226773 PMC3869884

[50] Verma R Lee C Jeun EJ Yi J Kim KS Ghosh A. Cell surface polysaccharides of *Bifidobacterium* bifidum induce the generation of Foxp3+ regulatory T cells. Sci Immunol. (2018) 3:eaat6975. 10.1126/sciimmunol.aat6975 30341145

[51] Song X Sun X Oh SF Wu M Zhang Y Zheng W. Microbial bile acid metabolites modulate gut RORγ(+) regulatory T cell homeostasis. Nature. (2020) 577:410–5. 10.1038/s41586-019-1865-0 31875848 PMC7274525

[52] Hang S Paik D Yao L Kim E Jamma T Lu J. Bile acid metabolites control TH17 and Treg cell differentiation. Nature. (2019) 576:143–8. 10.1038/s41586-019-1785-z 31776512 PMC6949019

[53] Campbell C McKenney PT Konstantinovsky D Isaeva OI Schizas M Verter J. Bacterial metabolism of bile acids promotes generation of peripheral regulatory T cells. Nature. (2020) 581:475–9. 10.1038/s41586-020-2193-0 32461639 PMC7540721

[54] Massoud AH Charbonnier LM Lopez D Pellegrini M Phipatanakul W Chatila TA. An asthma-associated IL4R variant exacerbates airway inflammation by promoting conversion of regulatory T cells to TH17-like cells. Nat Med. (2016) 22:1013–22. 10.1038/nm.4147 27479084 PMC5014738

[55] Mathias CB Hobson SA Garcia-Lloret M Lawson G Poddighe D Freyschmidt EJ. IgE-mediated systemic anaphylaxis and impaired tolerance to food antigens in mice with enhanced IL-4 receptor signaling. J Allergy Clin Immunol. (2011) 127:795–805.e6. 10.1016/j.jaci.2010.11.009 21167580 PMC3049834

[56] Noval Rivas M Burton OT Wise P Charbonnier LM Georgiev P Oettgen HC. Regulatory T cell reprogramming toward a Th2-cell-like lineage impairs oral tolerance and promotes food allergy. Immunity. (2015) 42:512–23. 10.1016/j.immuni.2015.02.004 25769611 PMC4366316

[57] Wohlfert EA Grainger JR Bouladoux N Konkel JE Oldenhove G Ribeiro CH. GATA3 controls Foxp3 + regulatory T cell fate during inflammation in mice. J Clin Invest. (2011) 121:4503–15. 10.1172/JCI57456 21965331 PMC3204837

[58] Noval Rivas M Burton OT Wise P Zhang Y Hobson SA Garcia Lloret M. A microbiota signature associated with experimental food allergy promotes allergic sensitization and anaphylaxis. J Allergy Clin Immunol. (2013) 131:201–12. 10.1016/j.jaci.2012.10.026 23201093 PMC3860814

[59] Delacher M Imbusch CD Weichenhan D Breiling A Hotz-Wagenblatt A Träger U Genome-wide DNA-methylation landscape defines specialization of regulatory T cells in tissues. Nat Immunol. (2017) 18:1160–72. 10.1038/ni.3799 28783152 PMC5912503

[60] Andreas N Potthast M Geiselhöringer AL Garg G de Jong R Riewaldt J. RelB deficiency in dendritic cells protects from autoimmune inflammation due to spontaneous accumulation of tissue T regulatory cells. J Immunol. (2019) 203:2602–13. 10.4049/jimmunol.1801530 31578269 PMC6826119

[61] Tordesillas L Berin MC Sampson HA Immunology of food allergy. Immunity. (2017) 47:32–50. 10.1016/j.immuni.2017.07.004 28723552

[62] Huang YJ Marsland BJ Bunyavanich S O'Mahony L Leung DYM Muraro A. The microbiome in allergic disease: current understanding and future opportunities-−2017 PRACTALL document of the American academy of allergy, asthma & immunology and the european academy of allergy and clinical immunology. J Allergy Clin Immunol. (2017) 139:1099–110. 10.1016/j.jaci.2017.02.007 28257972 PMC5899886

[63] Wilson MS Taylor MD Balic A Finney CA Lamb JR Maizels RM. Suppression of allergic airway inflammation by helminth-induced regulatory T cells. J Exp Med. (2005) 202:1199–212. 10.1084/jem.20042572 16275759 PMC2213237

[64] Navarro S Pickering DA Ferreira IB Jones L Ryan S Troy S. Hookworm recombinant protein promotes regulatory T cell responses that suppress experimental asthma. Sci Transl Med. (2016) 8:362ra143. 10.1126/scitranslmed.aaf8807 27797959

[65] de los Reyes Jiménez M Lechner A Alessandrini F Bohnacker S Schindela S Trompette A. An anti-inflammatory eicosanoid switch mediates the suppression of type-2 inflammation by helminth larval products. Sci Transl Med. (2020) 12:eaay0605. 10.1126/scitranslmed.aay0605 32321863

[66] Britton GJ Contijoch EJ Mogno I Vennaro OH Llewellyn SR Ng R. Microbiotas from humans with inflammatory bowel disease alter the balance of gut Th17 and RORγt+ regulatory T cells and exacerbate colitis in Mice. Immunity. (2019) 50:212–24.e4. 10.1016/j.immuni.2018.12.015 30650377 PMC6512335

[67] Al Nabhani Z Dulauroy S Marques R Cousu C Al Bounny S Déjardin F. A weaning reaction to microbiota is required for resistance to immunopathologies in the adult. Immunity. (2019) 50:1276–88.e5. 10.1016/j.immuni.2019.02.014 30902637

[68] Ramanan D Sefik E Galván-Peña S Wu M Yang L Yang Z. An immunologic mode of multigenerational transmission governs a gut Treg setpoint. Cell. (2020) 181:1276–90.e13 10.1016/j.cell.2020.04.030 32402238 PMC7393667

